# Protection of Pepper Plants from Drought by *Microbacterium* sp. 3J1 by Modulation of the Plant's Glutamine and α-ketoglutarate Content: A Comparative Metabolomics Approach

**DOI:** 10.3389/fmicb.2018.00284

**Published:** 2018-02-22

**Authors:** Juan I. Vílchez, Karsten Niehaus, David N. Dowling, Jesús González-López, Maximino Manzanera

**Affiliations:** ^1^Department of Microbiology, Institute for Water Research, University of Granada, Granada, Spain; ^2^Proteom- und Metabolomforschung, Fakultät für Biologie, Centrum für Biotechnologie, Universität Bielefeld, Bielefeld, Germany; ^3^Department Science & Health, Institute of Technology Carlow, Carlow, Ireland

**Keywords:** PGPR, *Microbacterium* sp. 3J1, *Capsicum annum*, trehalose, desiccation-tolerance, RDTE, comparative metabolomics

## Abstract

Drought tolerance of plants such as tomato or pepper can be improved by their inoculation with rhizobacteria such as *Microbacterium* sp. 3J1. This interaction depends on the production of trehalose by the microorganisms that in turn modulate the phyto-hormone profile of the plant. In this work we describe the characterization of metabolic changes during the interaction of pepper plants with *Microbacterium* sp. 3J1 and of the microorganism alone over a period of drought. Our main findings include the observation that the plant responds to the presence of the microorganism by changing the C and N metabolism based on its glutamine and α-ketoglutarate content, these changes contribute to major changes in the concentration of molecules involved in the balance of the osmotic pressure. These include sugars and amino-acids; the concentration of antioxidant molecules, of metabolites involved in the production of phytohormones like ethylene, and of substrates used for lignin production such as ferulic and sinapic acids. Most of the altered metabolites of the plant when inoculated with *Microbacterium* sp. 3J1 in response to drought coincided with the profile of altered metabolites in the microorganism alone when subjected to drought, pointing to a response by which the plant relies on the microbe for the production of such metabolites. To our knowledge this is the first comparative study of the microbe colonized-plant and microbe alone metabolomes under drought stress.

## Introduction

Drought negatively affects the physiology and biochemistry of plants. Many mechanisms have been described in response to drought which include the synthesis of compatible solutes for example, amino acids such as proline and betaine. In addition quaternary compounds, amines and different types of sugars, such as trehalose and the raffinose family oligosaccharides (RFOs) have also been implicated (Mahajan and Tuteja, 2005; Julca et al., 2012). An increase in trehalose concentration, in addition to its xeroprotective effects, also modulates the phytohormone profile of the plant (Rodriguez-Salazar et al., 2009; Yang et al., 2010; Lunn et al., 2014). This accumulation of compatible solutes is normally accompanied by the accumulation of antioxidant molecules to maintain cell turgor pressure, and to protect essential biomolecules from the cell, including membranes and enzymes from oxidative damage (Gill and Tuteja, 2010; Krasensky and Jonak, 2012; Gagné-Bourque et al., 2016).

Other mechanisms proposed for the protection of plants against drought includes the so-called induced systemic tolerance (IST) process. This process involves the modification of phytohormonal profiles, production of antioxidant defense mechanisms, the use of bacterial exopolysaccharides (EPS), and those associated with accumulation of several xeroprotectants such as sugars, amino acids, and polyamines by associated microorganisms. In addition, the production of heat-shock proteins (HSPs), dehydrins, and volatile organic compounds (VOCs) are involved in the acquisition of drought tolerance (Kaushal and Wani, 2016; Vurukonda et al., 2016).

The concentration of the gas phytohormone ethylene increases in the plant during drought stress and results in smaller plant roots and shoots, and might induce gene expression for fruit ripening and for inhibition of seed germination as well as for the senescence and abscission of leaves (Lincoln and Fischer, 1988; Abeles et al., 1992). The biosynthetic pathway for ethylene under water stress in the plant occurs via Yang's cycle, where three enzymatic steps are involved. Firstly, the conversion of methionine (Met) to S-adennosyl-L-methionine (SAM), then the transformation of SAM into 1-aminocyclopropane-1-carboxylic acid (ACC) and finally the conversion of ACC into ethylene. Under non-stressing conditions Met is recovered by production of 5-methylthioadenosine (MTA) from SAM, and then MTA is transformed into 5'-Methylthioribose that is subsequently phosphorylated to 5'-Methylthioribose-1-phosphate and from this into 2-keto-4-methylthiobutyrate (KMTB), and finally KMTB is transformed into Met (Sauter et al., 2013). Certain microorganisms synthesize the enzyme ACC deaminase that cleaves the ethylene precursor ACC to ammonia and α-ketobutyrate, and consequently lowers the ethylene level and its detrimental effect on the plant (Glick et al., 1998; Penrose and Glick, 2001; Shaharoona et al., 2006). Ethylene is also a key regulator for the bacterial colonization and persistence in the plant and therefore regulation of ethylene production is most likely mediated by its effect on the plant signaling pathways (Hardoim et al., 2008). Bacteria are able to modulate plant ethylene levels either by cleaving ACC (Glick et al., 2007), or by inhibiting ACC synthase and/or β-cystathionase, both enzymes of the ethylene biosynthesis pathway (Sugawara et al., 2006).

Bacteria able to protect plants from drought are termed Rhizobacteria Drought-Tolerance Enhancers (RDTE), although mechanisms to protect plants from drought are not restricted to the ethylene metabolism, production of compatible solutes, antioxidant molecules, or phytohormones. Water stress also causes changes in the lignin composition of the plant. Lignin which is a phenolic polymer complex is derived mainly from three hydroxycinnamoyl alcohols (monolignol *p*-coumaryl, coniferyl, and sinapyl) (Boerjan et al., 2003; Ralph et al., 2004). Alvarez et al. (2008) determined that *p*-coumaric acid and caffeic acid concentration increased in maize xylem sap of plants subjected to water stress, while ferulic acid concentration decreased under similar conditions (Alvarez et al., 2008). These compounds are involved in regulating the relative abundance of the monolignols that may be polymerized into lignin, and therefore, these authors suggested that this accumulation was due to a decrease in lignin biosynthesis (Alvarez et al., 2008). In addition, the accumulation of such intermediates in response to water stress seems to be related to the drought tolerance of the plant since different varieties of barley accumulate concentrations of *p*-coumaric, sianpic, and cinnamic acids differently in response to drought (Chmielewska et al., 2016). It is also worth mentioning that the production and exudation of *p*-coumaric acid by the plant decreases the microbial biodiversity and abundance of bacteria and fungi in the rhizosphere (Zhou and Wu, 2012).

Using a collection of actinobacteria as RDTE we have recently described that these microorganisms can interact with tomato and pepper plants affecting their phytohormone profile (Vílchez et al., 2016). The protection of the plants seems to be correlated with the concentration of trehalose produced by the microorganisms that in turns alters the metabolism of the plant. Among the collection of microorganisms *Microbacterium* sp. 3J1 showed the highest production of trehalose and plants inoculated with these bacteria showed the highest relative water content, fresh, and dry weight during drought periods of up to 33 days.

In this study, we investigated the modulation of metabolites expressed in pepper plants colonized by *Microbacterium* sp. endophytic strain 3J1 during drought conditions and compared those changes with the ones occurring in the bacterium alone in response to the same stress. To our knowledge there is no information on comparative metabolomics of inoculated plants and their corresponding RDTE alone in response to drought. We also explored the physiological responses of pepper plants under drought stress in association with *Microbacterium* sp. 3J1. To that end we focused on the analysis of the fluctuation of sugars, amino acids, and organic acids produced by the plant in response to drought in presence and absence of *Microbacterium* sp. 3J1. We compared this metabolite profile with the metabolites produced by the microorganism alone to find out if the metabolites overexpressed by the plant might proceed from *Microbacterium* sp. 3J1 subjected to drought conditions.

## Materials and methods

### Microorganisms, media, and culture conditions

*Microbacterium* sp. 3J1 strain was used in this study as an RDTE (Narváez-Reinaldo et al., 2010). Bacteria were grown in tryptic soy broth (TSB) at 30°C (Manzanera et al., 2004). To generate hyperosmotic conditions, 5 or 50% (wt/vol) polyethylene glycol (PEG) 6000 was added to the bacterial media (Sandhya et al., 2009) or watering was stopped for plants.

### Construction of the fluorescent *Microbacterium* sp. 3J1-GFP strain

To generate a fluorescent version of *Microbacterium* sp. 3J1, a tetraparental mating method was used following the method described by Lambertsen et al. (2004). As donor and helper strains, *Escherichia coli* GFP2 Gm^R^ and *E. coli* pUX-BF13 Ap^R^ were employed, respectively. Finally, the strain *E. coli* pRK600 Cm^R^ was employed to mobilize the other plasmid in the mating process. After 4 h mating, tagged-bacterium selection was carried out in Gm_30_ containing LB plates after a 24 h drought period and re-streaking of single colonies to perform an insertion test by PCR.

### Plant material and growth conditions

The drought-sensitive green pepper (*Capsicum annuum* L. *cv*. Maor) seedlings were germinated from sterile seeds that were sown in plastic trays in wet vermiculite accordingly to Mayak et al. (2004) and Vílchez et al. (2016). After 2 weeks, uniform-sized plants (shoot height ~5 cm) were selected and planted in non-sterile soil composed of a mixture of plant substrate (black peat, vegetable compost, white peat, and coconut bark pH 7.2 and 56% organic matter) and non-sterile vermiculite (1:1 v/v), one per pot, using 0.4 L pots filled with ~0.26 L of soil mixture. Alternatively, 1 month-old plants were purchased from SaliPlant S.L. specialist grower (Granada, Spain). The pots were incubated in a growth room at constant relative humidity (50–60%). The room was lit with a 12-h day/night cycle and gradual dimming/brightening of the light to simulate dawn and dusk. The day cycle consisted of 200 μmol photons·m^−2^·s^−1^, and the dawn–dusk cycle consisted of 150 μmol photons·m^−2^·s^−1^. The temperature was programmed to change from 18 to 20°C for the night cycle to 20–25°C in the diurnal cycle. Plants were regularly watered for 5 to 15 days.

At inoculation, 30 seedlings were treated with 40 mL of bacterial suspension (10^8^-10^9^ CFU/mL) in sterile M9 buffer, and non-inoculated controls were watered with sterile M9 buffer.

#### Photosystem activity efficiency

Photosystem activity efficiency was recorded as the Qy parameter (equivalent to Fv/Fm) by using a FluorPen 100 (PSI, Photon Systems Instruments, Brno, Czech Republic) with at least nine measures per seedling and at least five seedlings per condition.

#### Confocal fluorescent microscopy

A detailed analysis of samples was carried out by using an inverted confocal laser-scanning microscope Leica DMI6000 with a laser from blue diode 405 nm, Argon 458-514 nm to He/Ne 543-633 nm (Center for Scientific Instrumentation, UGR, Granada, Spain). After identifying and detailing the visual fields, the options that allow taking sequences and quality point shots were adjusted with the Leica Software LAS X (Leica Microsystems, Wetzlar, Germany. With layer sequences in different depths, transdimensional confocal-combined images were assembled using the same software. Objectives of 40-100X with immersion oil were required to acquire the images.

### Quantitative PCR

In order to identify and quantify the number of copies of the *gfp* gene from both DNA extracted from the soil and that obtained from roots or other plant tissues, a quantitative real-time PCR (qRT-PCR) was performed according to the instructions of Liu et al. (2010) with some modifications (Couillerot et al., 2010; Liu et al., 2010; Quecine et al., 2012). DNA extraction was performed after 3 h of inoculation and every 24 h for 14 days.

In order to estimate the number of copies, DNA curves were prepared from pure DNA digests extracted from cultures whose concentrations were previously determined with a NanoDrop 2000 kit (Fisher Scientific, USA). Calibration curves were used as the quantification pattern for the DNA extracted from each of the inoculated soils.

The qRT-PCR was performed with the LightCycler Nano team (Roche Life Science, USA) using the commercial FastStart essential DNA Green Master kit (Roche) with a Master Mix preparation with SYBRGreen as a fluorescent intercalating agent.

Master mixtures contained 6 μL of PCR quality H_2_O, 10 μL of the commercial mixture (containing 10X buffer, 200 μM dNTPs, 1x SYBER Green and 3 mM MgCl_2_), 1 μL of each primer, and 2 μL of template DNA in a final volume of 20 μL. The program for real-time amplification and quantification was as follows: initial denaturation of 95°C for 5 min, amplification phase of 45 cycles with a denaturation phase of 95°C (30 s), a phase annealing at 58°C (15 s), and one of denaturation at 72°C (30 s), melting curve with a single cycle of 95°C (5 s), 65°C (1 min), and 95°C (5 s), and completion with 37°C (30 s). The fluorescence intensity acquisition was recorded at the end of each banding cycle.

### Quantification of number of microbial cells by culture-dependent methods

To quantify the number of recoverable or viable cells as colony forming units (CFUs), plate seeding method was carried out after a serial dilution from original sample. Regular tryptic-soy agar plates (TSA) or selective antibiotic-containing TSA plates were employed to perform the quantifications in each case. Plates were incubated at 30°C for 48 h to allow a recommended growth of the strain.

### Ethylene production

Ethylene production by the plants was quantified as described by Yang and Hoffman (1984). Plants were introduced in 30 mL glass vials sealed with a rubber stopper and aluminum ring. Produced ethylene was measured was determined using a GC HP5890, fitted with a flame ionization detector with a mixture o air (2 bars) and hydrogen (1 bar) and a 180 cm length poropak column (3.2 mm diameter section). Nitrogen was used as carrier gas at a 55 mL/min flow. Column was heated at 65°C, injector at 120°C, and detector worked at 105°C.

### Plant and bacteria sampling. metabolomics

After 33 days of drought stress, for plants, at least 0.5 g of the drought stressed plants and controls were harvested. At least three plants for each condition were sampled and washed twice with distilled water before roots and shoots were harvested always 2 h after the onset of day cycle. For each bacterial treatment, 50 mL of a TSB culture of the strain (1.0 OD 600 nm) were freeze-dried to obtain a dry powder. Samples were directly frozen in liquid nitrogen and stored at −80°C until further processing. Samples were homogenized using micropistils in 1.5 mL tubes. Extraction and derivatization was performed as described by Roessner et al. (2000) with minor amendments based on Barsch et al. (2004).

From the homogenized samples, 10–30 mg of dry samples were taken and added into 1 mL of 80% methanol with ribitol (1 μM, as an internal standard) in 1.5 mL tubes with 0.5 g of acid-washed glass/ceramic beads with a diameter not greater than 0.5 mm (Müller et al., 2015). Then the samples were homogenized immediately three times at 6.5 m/s for 45 s using a FastPrep homogenizer (MP Biomedicals, USA). Samples were incubated at 70°C for 15 min and then were centrifuged for 20 min at 15,000 × g at room temperature. Part of the clear supernatant (375 μL) was transferred to 1 ml glass vials (Reactivials, Supelco, Bellefonte, California) and evaporated in a dry nitrogen stream. Metabolite functional groups were derivatized by addition of 100 μL methoxylamine hydrochloride in pyridine (20 mg/mL; g/v) for 90 min at 37°C and with 100 μL MSTFA for 30 min at 37°C. Samples were constantly mixed by a micro-stirring bar. All chemicals and standard compounds were purchased from Sigma–Aldrich-Fluka (Taufkirchen, Germany), Merck (Darmstadt, Germany), or Macherey-Nagel (Düren, Germany).

### GC-MS analysis

A TraceGC gas chromatograph was used to analyze sample volumes of 1 μL. The chromatograph was coupled to a PolarisQ ion trap mass spectrometer (both Thermo Finnigan, Dreieich, Germany). Using an AS2000 auto sampler (Thermo Finnigan), derived metabolites were injected and evaporated at 250°C in splitless mode and separated on a 30 m × 0.25 mm Equity-5 column with 0.25 μm coating of 5% diphenyl 95% dimethylsiloxane (Supelco, Bellefonte, California, USA). Helium carrier-gas flow was adjusted to 1 ml/min. The interphase temperature was set to 250°C and the ion source temperature to 200°C. Oven temperature was kept constant for 3 min at 80°C and subsequently raised to 300°C at 3°C/min. To equilibrate the system an incubation of 2 min at 80°C was applied after each analysis. Mass spectra were recorded at 2 scan/s with a scanning range of 50 to 550 m/z. Metabolites were identified by comparison with purified standards, the NIST 2005 database (NIST, Gaithersburg, MD, USA) (Nist-Database, 2005) and the Golm Metabolome Database (Kopka et al., 2005). All identified metabolites matched the references by mass spectral data and chromatographic retention time. These selected metabolites peak areas were automatically quantified using the processing setup implemented in the Xcalibur 1.4 software (Thermo Finnigan, Dreieich, Germany). The relative response ratios calculated from the peak areas were normalized by the internal standard ribitol and dry mass of the sample. Experiments were performed with six replicates, which consisted of three independent biological replicates and three technical replicates.

### Data analyses and visualization

Pirouette 3.02 (Infrometrix, Woodinveille, Wash, USA) software was used to perform the principal component analysis (PCA) (Fiehn et al., 2000). For heatmaps and statistics, data were log10-transformed and centered (van den Berg et al., 2006). For root metabolites, one-way-ANOVA and Tukey's test (*P* ≤ 0.05) were performed with SigmaPlot 11 (Systat Software, San Jose, USA). Heatmaps were created with the MultiExperiment Viewer (MeV 4.9, http://www.tm4.org) by using Pearson's correlation and complete linkage. For the metabolic map, untransformed mean values were employed to calculate –P/+P ratios.

## Results and discussion

### *Microbacterium* sp. 3J1 colonizes pepper plants as an endophyte

**Figure 1:**
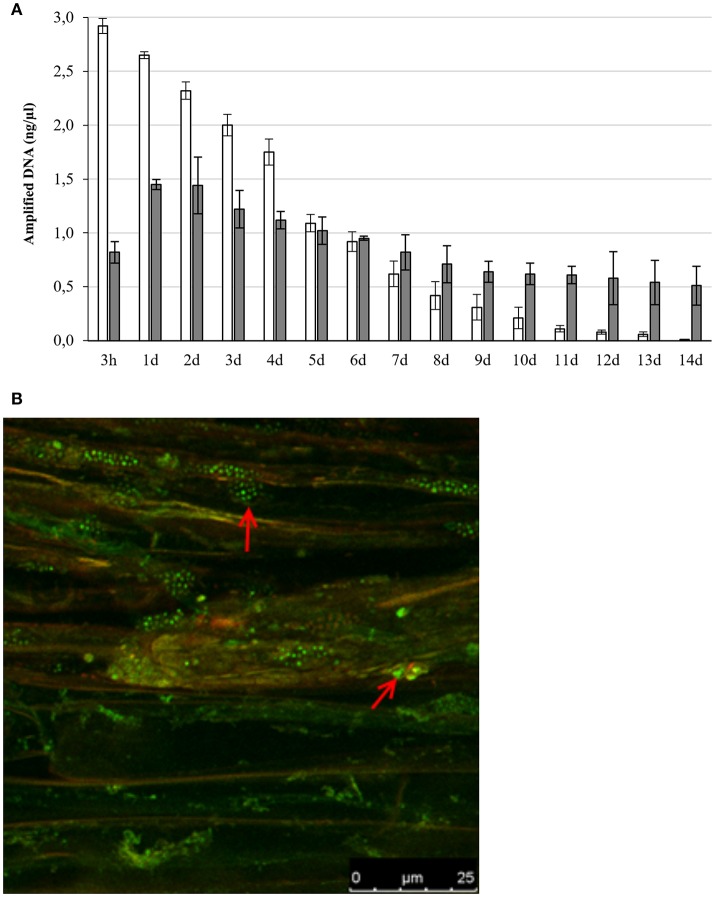
Presence of *Microbacterium* sp. 3J1-GFP in soil and plant tissue. Panel **(A)** represents the concentration of *gfp* gene DNA specifically amplified by qRT-PCR over a 14-day period from soil (white bars) and plant tissue (gray bars). Panel **(B)** shows the colonization of pepper roots by fluorescent confocal microscopy (red arrows indicate the bacterial colonization).

In the interaction between plants and microorganisms the bacteria must first establish contact with the plant root, overcome the plant defense system before promoting plant health and growth by colonizing the inside the plant (Truyens et al., 2015; Gagné-Bourque et al., 2016). To determine the different steps needed for the interaction between *Microbacterium* sp. 3J1 and pepper plants we inserted the *gfp* gene coding for the Green Fluorescent Protein (GFP) into *Microbacterium* sp. 3J1. This generated a fluorescent version of the strain termed *Microbacterium* sp. 3J1-GFP by transposition of the marker using a tetraparental mating as described in Materials and Methods. The detection of the bacteria in soil determined by qPCR declined with time, while internal and systemic colonization of pepper plants by *Microbacterium* sp. 3J1-GFP was observed over the 14-day period (Figure 1A). In addition, the number of cells in soil was determined by culture dependent methods showing similar results. The presence of *Microbacterium* sp. 3J1-GFP in root tissues of the plant under water stressed and non-stressed plants, was determined by confocal fluorescent microscopy identifying the microorganisms inside the root tissues, showing that the microbial strain efficiently colonized the rhizosphere and pepper roots and that it was also intimately associated with the plant since it was isolated from the interior of root and shoot tissues of inoculated plants (Figure 1B).

### *Microbacterium* sp. 3J1 improves the physiology of water-stressed plants

**Figure 2:**
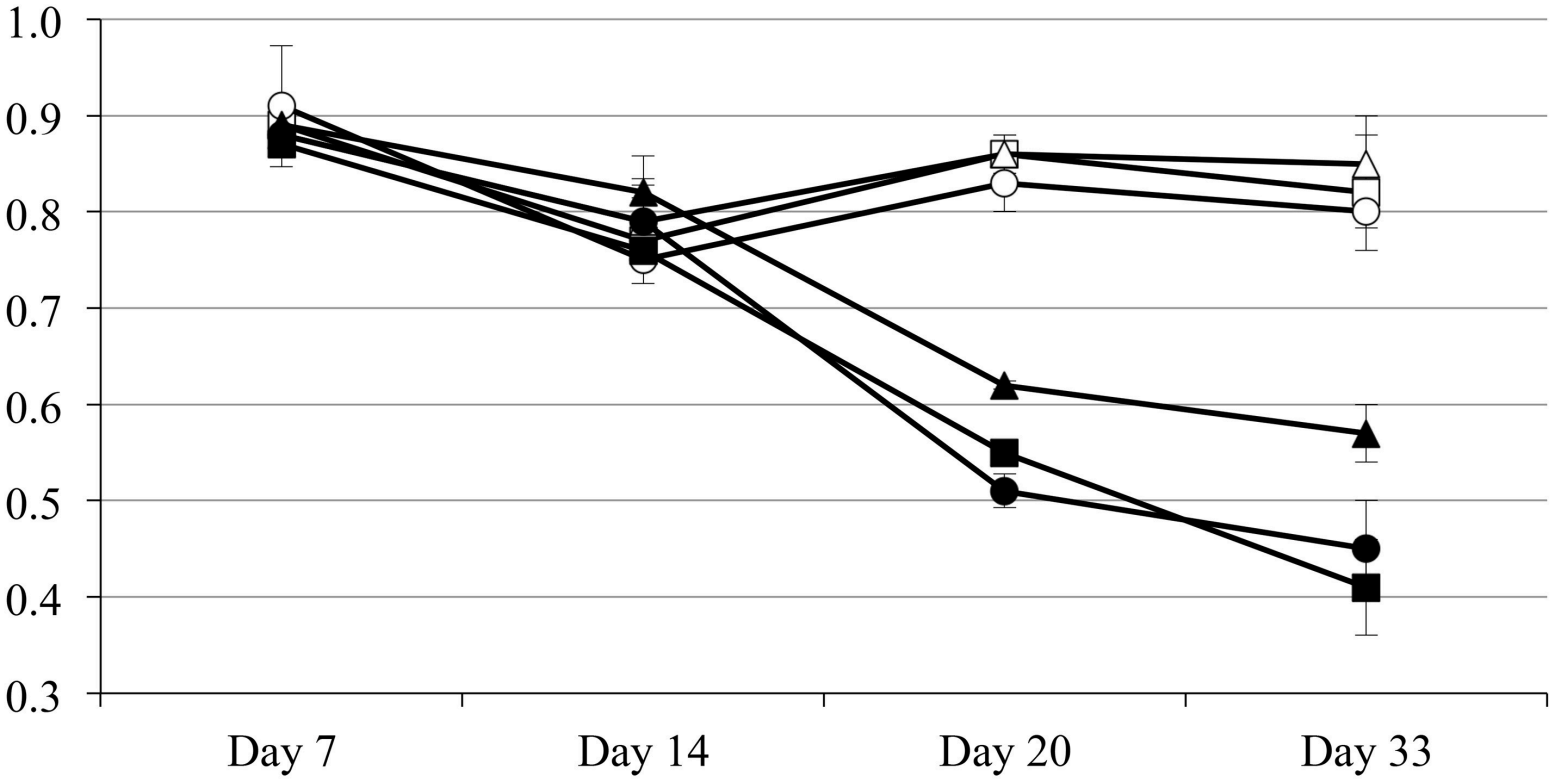
Relative water content of inoculated and non-inoculated plants of different ages. Five-day old (squares), 10-day old (circles), and 15-day old (triangles) plants inoculated with *Microbacterium* sp. 3J1 (open symbols) and non-inoculated (solid symbols).

Plants subjected to drought respond by reducing their stomatal conductance to minimize the water loss due to transpiration (Medrano et al., 2002). The main drawback in this physiological response is the reduction in CO_2_ availability and the concomitant reduction on the plant photosynthetic rate, as well as the accumulation of reactive oxygen species (ROS) and therefore the incidence of oxidative damage (Arbona et al., 2017). Therefore, the plant needs to find a compromise between carbon fixation and water loss due to transpiration. To determine the physiological response to drought of pepper plants inoculated with the endophyte *Microbacterium* sp. 3J1 the relative water content (RWC) and photosystem II efficiency were measured. With regard to the RWC, plants in absence of watering inoculated with *Microbacterium* sp. 3J1 showed similar RWC within the 33-day trial independently of their age when using 5, 10, and 15-day old plants (with fresh weight of 31, 245, and 873 mg). However, the RWC of these plants decreased over the 33-day drought when incubated in absence of the 3J1 strain, although the reduction of the RWC was less pronounced with older plants (Figure 2). This indicates that the interaction between the microorganism and the plant is independent of the plant age or size for the first 15 days.

**Figure 3:**
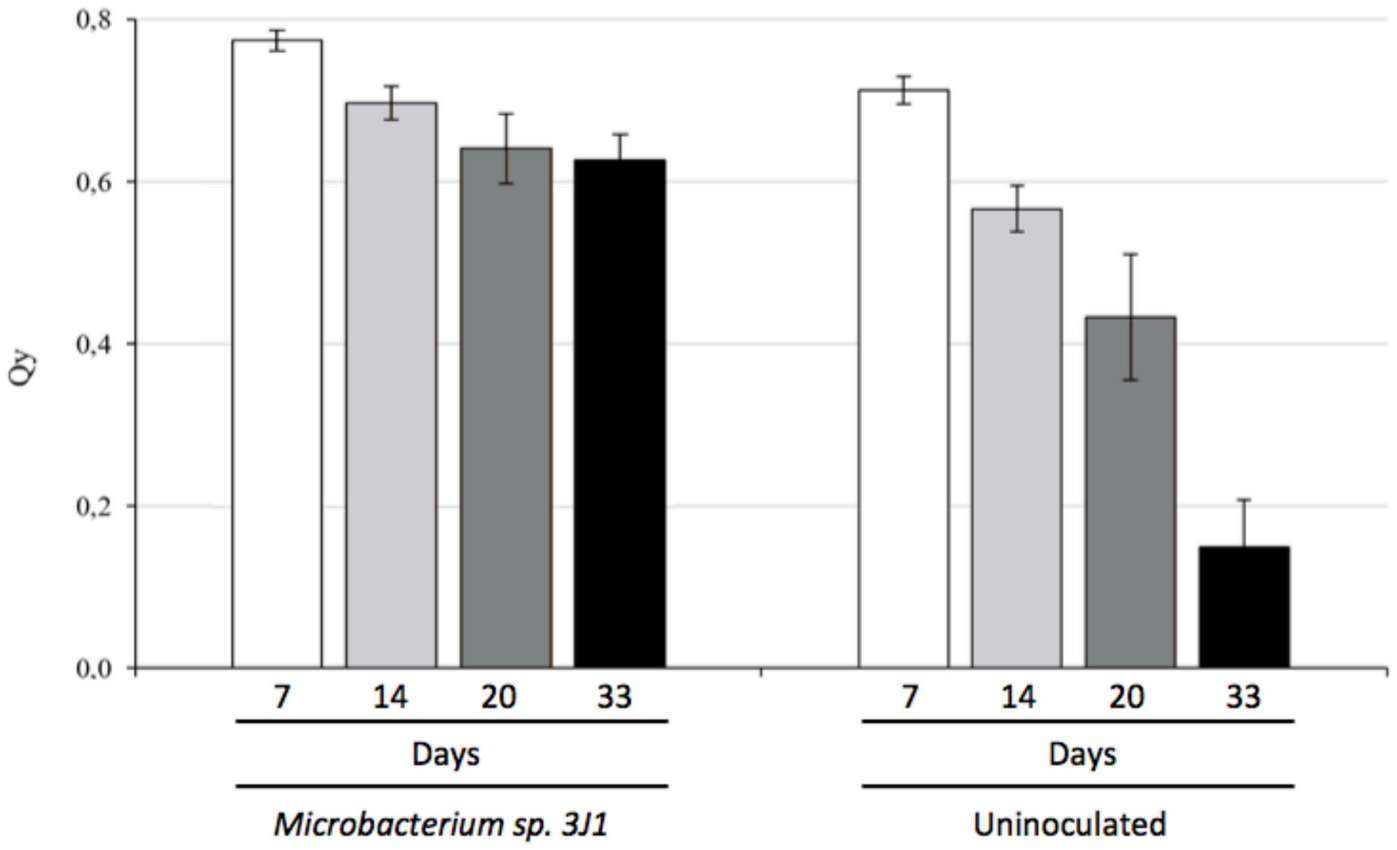
Photosynthesis efficiency of pepper plants subjected to drought and measured as Qy (Fv/Fm). White bars show Qy at day 7, light-gray bars at day 14, dark-gray bars correspond to day 20 and black bars to 33 days in absence of watering.

**Figure 4:**
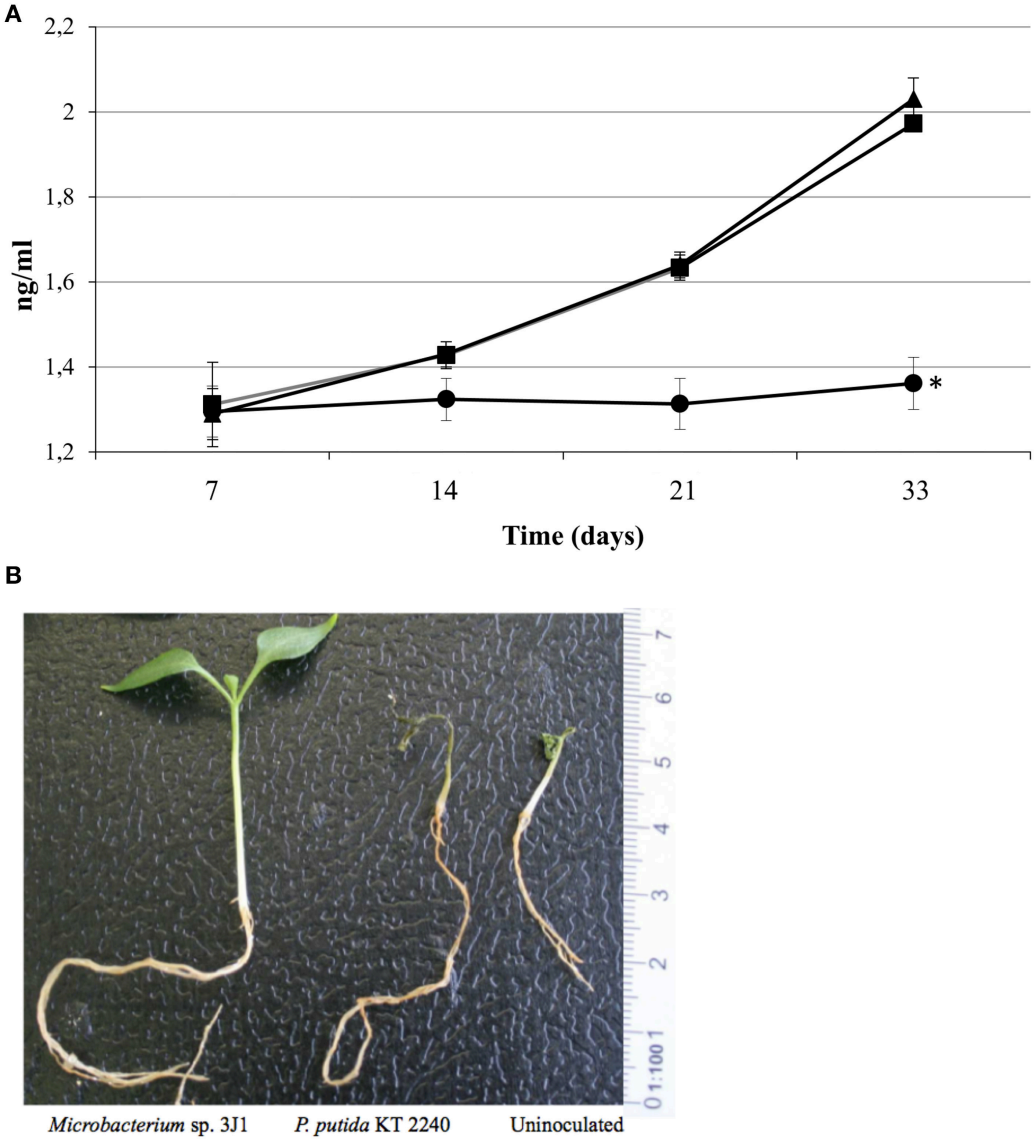
Ethylene produced by inoculated and non-inoculated plants and their aspect. Ethylene produced (in ng/ml) by plants inoculated with *Microbacterium* sp. 3J1 (circles) and with the desiccation sensitive microorganism *P. putida* KT2440 (squares) as well as by non-inoculated plants (triangles) **(A)**. And aspect of plants after 33 days of drought **(B)**.

Photosynthesis efficiency is a primary indicator under stress conditions due to chloroplast function being quite sensitive to lack of water or water restrictions. When we measured the Qy parameter (equivalent to Fv/Fm and ranging from 0 to 1) we also observed that pepper plants inoculated with *Microbacterium* sp. 3J1 showed higher photosynthesis efficiency (between 0.8 at day 7 and 0.6 after 33 days of drought) under drought conditions than non-inoculated plants, with a Qy that sharply decreased to values close to 0.1 after 33 days under drought conditions (Figure 3). In addition, endophyte-inoculated pepper plants under drought conditions produced a much lower concentration of ethylene (Figure 4A) that might explain the reduced senescent aspect of the plants, compared with the non-inoculated plants (Figure 4B). Reduction of ethylene production under water-stress conditions is one of the most commonly reported plant responses mediated by PGB inoculation in various crops (Glick et al., 2007; Glick, 2014; Müller et al., 2015). This reduction of ethylene production seems to be meditated among other factors by the production of ACC-deaminase by the microorganism as we have described previously (Vílchez et al., 2016) although we do not discard other mechanisms as those described in the Introduction.

### Metabolic response of inoculated pepper plants to drought stress

#### Analysis overview

The biochemical strategies that plants use to resist drought include the production and accumulation of compatible solutes as well as other key metabolites (Krasensky and Jonak, 2012; Arbona et al., 2017). To determine the differences in the metabolites accumulated by *Microbacterium* sp. 3J1-inoculated pepper plants with non-inoculated plants under drought conditions, we analyzed the differences in metabolite accumulation in both conditions in roots (Table 1). These analyses showed important differences in the metabolite profile between inoculated and non-inoculated plants under drought conditions.

**Table 1A:** Metabolites concentrated in inoculated roots compared to non-inoculated ones by chemical groups and drought response function.

**Metabolite**	**Ratio**	**Type**
Alanine^a^^,^^c^^,^^d^	3.96	Amino acid
Valine^a^^,^^d^	49.08	Amino acid
Leucine^a^^,^^d^	3.04	Amino acid
Isoleucine^b^^,^^c^	5.76	Amino acid
Proline^a^^,^^b^^,^^e^	2.80	Amino acid
Tryptophan^a^^,^^c^	1.48	Amino acid
Glutamine^b^^,^^d^	456.60	Amino acid
Ornithine-Citruline-Arginine^a^	39.79	Amino acid
Asparagine^a^	27.64	Amino acid
Arginine NH_3_^a^	1.70	Amino acid
1-metil-L-histidina^c^^,^^f^	6.20	Amino acid
Tyrosine^b^^,^^c^	1.60	Amino acid
L-Cystathionine^c^	2.10	Amino acid
Spermidine^c^^,^^e^	7.65	Polyamine
N-Acetylglucosamine^a^^,^^b^	10.84	Amino Sugar
Ethanolamine^a^^,^^c^	29.69	Amino Alcohol
Adenosine^c^^,^^d^	2.45	Nucleoside
Apigenine^b^	3.79	Flavon
(-)-Epicatechin^b^	7.92	Flavonoid
Daidzein^b^^,^^c^^,^^d^	5.37	Isoflavon
Uracil^a^^,^^d^	4.57	Nitrogenous base
Thymine^b^^,^^c^	2.56	Nitrogenous base
Urea^a^^,^^c^	2.91	Nitrogenous compound
Pyruvate^a^	2.02	Organic acid
Malate^a^^,^^c^	5.35	Organic acid
Cis-aconitate^a^^,^^d^	1.72	Organic acid
Isocitrate^a^^,^^b^	4.34	Organic acid
Shikimate^a^^,^^d^	1.73	Organic acid
2 Methylcitrate^a^	1.65	Organic acid
Gluconate^a^	3.72	Organic acid
Glucuronic acid^a^^,^^b^^,^^c^^,^^e^	3.51	Organic acid
Sinapic acid^c^^,^^e^	2.85	Organic acid
Caffeic acid^b^^,^^c^^,^^d^^,^^e^	3.94	Organic acid
Dihydrocaffeic acid^b^^,^^d^	2.16	Organic acid
Quinic acid^b^	1.49	Organic acid
Maloic acid^a^^,^^b^	2.31	Organic acid
Ferulic acid^b^^,^^e^	1.89	Organic acid
Siring Aldehyde^e^	3.24	Aldehyde
Conifer Aldehyde^d^^,^^e^	20.43	Aldehyde
trans-Chalcone^b^	4.64	Ketone
Myo-inositol^a^	5.15	Polyalcohol
Mannitol^a^	3.05	Polyalcohol
Glycerol-3-P^c^^,^^d^	1.69	Phosphated compound
Glycerate-3-P^c^^,^^d^	2.80	Phosphated compound
Fructose-6-P^c^^,^^d^	17.94	Phosphated compound
Glucose-6-P^a^^,^^c^	10.87	Phosphated compound
Gluconate-6-P^a^	30.39	Phosphated compound
Ribose-5-P^a^^,^^d^	7.07	Phosphated compound
Xylose^a^^,^^d^^,^^e^	1.92	Sugar
Fructose^a^^,^^d^	8.12	Sugar
Galactose^a^	3.61	Sugar
Melibiose^a^^,^^d^	41.87	Sugar
Maltose^a^^,^^d^	4.65	Sugar
Celobiose^a^^,^^d^	2.49	Sugar
Trehalose^a^^,^^c^^,^^e^	84.79	Sugar
Raffinose^a^^,^^b^	2.01	Sugar
Melicitose^a^	2.34	Sugar

*The ratio between the detected concentrations in each condition is also included*.

a *Osmotic balance*;

b *Anti-ROS*;

c *Phytohormone/Ethylene Cycle*;

d *Signaling*;

e *Structural*;

f *Other*.

**Table 1B:** Metabolites which concentration decreases in *Microbacterium* sp. 3J1-inoculated pepper plants by chemical groups and drought response function.

**Metabolite**	**Ratio**	**Type**
Cysteine^b^	23.81	Amino acid
Serine^d^	18.02	Amino acid
S-methylcysteine^c^	9.39	Amino acid
ß-alanine^a^	1.74	Amino acid
Methionine^c^	5.88	Amino acid
Histidine^a^^,^^c^^,^^f^	1.44	Amino acid
Cystine^b^	2.29	Amino acid
4-aminobutirate (GABA)^a^^,^^c^^,^^d^	1.95	Amino acid
L-Aspartate^b^^,^^c^	1.53	Amino acid
Allantoin^c^	1.57	Nitrogenous compound
Sinapaldehyde^e^	1.91	Aldehyde
Naringenin^b^^,^^c^^,^^d^	1.71	Isoflavonoid
Coumesterol^b^^,^^c^	2.18	Isoflavon
Cinnamic acid^b^^,^^c^	1.80	Organic acid
p-Coumaric acid^c^^,^^f^	9.36	Organic acid
Gluconic acid^b^^,^^d^^,^^e^	5.78	Organic acid
ß-aminoisobutyric acid^a^^,^^d^^,^^e^	10.13	Organic acid
Glycerate^a^^,^^b^^,^^d^	5.94	Organic acid
Ketoglutarate^a^^,^^b^	70.98	Organic acid
2-hydroxiglutarate^b^	3.98	Organic acid
2-aminoadipate^a^	3.81	Organic acid
Phosphoenolpyruvate (Pep)^b^^,^^d^	2.48	Organic acid
Gluconate-1,5-lactone^f^	1.87	Organic acid
Erythrose-4-P^a^	1.59	Phosphated compound
Ribulose-5-P^b^^,^^d^	4.33	Phosphated compound
Ribose^c^^,^^d^	3.35	Sugar

*The ratio between the detected concentrations in each condition is also included*.

a Osmotic balance;

b Anti-ROS;

c Phytohormone/Ethylene Cycle;

d Signaling;

e Structural;

f *Other*.

##### Sugars

Carbohydrate metabolism is profoundly affected by drought with a general degradation of starch in the chloroplasts of osmotically stressed tissues to sustain both carbon export and osmolyte accumulation in different tissues (Thalmann et al., 2016). Reduction in sucrose concentration was found probably due to an induced increase of invertase activity (Ruan et al., 2010). Drought stressed plants normally accumulate soluble sugars such as, sucrose, glucose, fructose, raffinose, and other RFOs to adjust the osmotic balance and to procure the needed turgor pressure under water scarcity (Krasensky and Jonak, 2012; Zanella et al., 2016). Gagné-Bourque et al. (2016) found that bacterized Timothy (*Phleum pratense* L.) plants with *Bacillus subtilis* B26 subjected to drought generally accumulated higher levels of sugars most notably sucrose and fructans (Gagné-Bourque et al., 2016). In this report, we identified trehalose as the most affected sugar in pepper plants subjected to drought by the presence of *Microbacterium* sp. 3J1 with nearly an 85-fold increase, followed by melibiose with nearly a 42-fold increase. Trehalose has been reported as one of the most efficient protectants against drought especially in desiccation tolerant plants such as the Resurrection Plant (*Selaginella lepidophylla*) (Yobi et al., 2013). We speculate that the increase of trehalose in the plant when inoculated with 3J1 strain might also lead to cross-talk with ABA signaling for stomata closure since trehalose-6-P has already has being described for ABA-dependent germination by Gómez et al. (2010). Melibiose in conjuction with fructose are the hydrolytic products of raffinose that can be further hydrolyzed into galactose and glucose, all of these are accumulated by the plant under drought when in presence of the 3J1 strain. Then other sugars such as maltose, cellobiose, melicitose (a non-reducing tri-saccharide), or xylose were also found to increase in concentration from ~ 8- to 2-folds. These results show that the presence of *Microbacterium* sp. 3J1 contributed to the increase in the biosynthesis of sugars which allows for better osmotic adjustment and thus alleviates the stress effect on the host plant.

##### Amino acids

**Figure 5:**
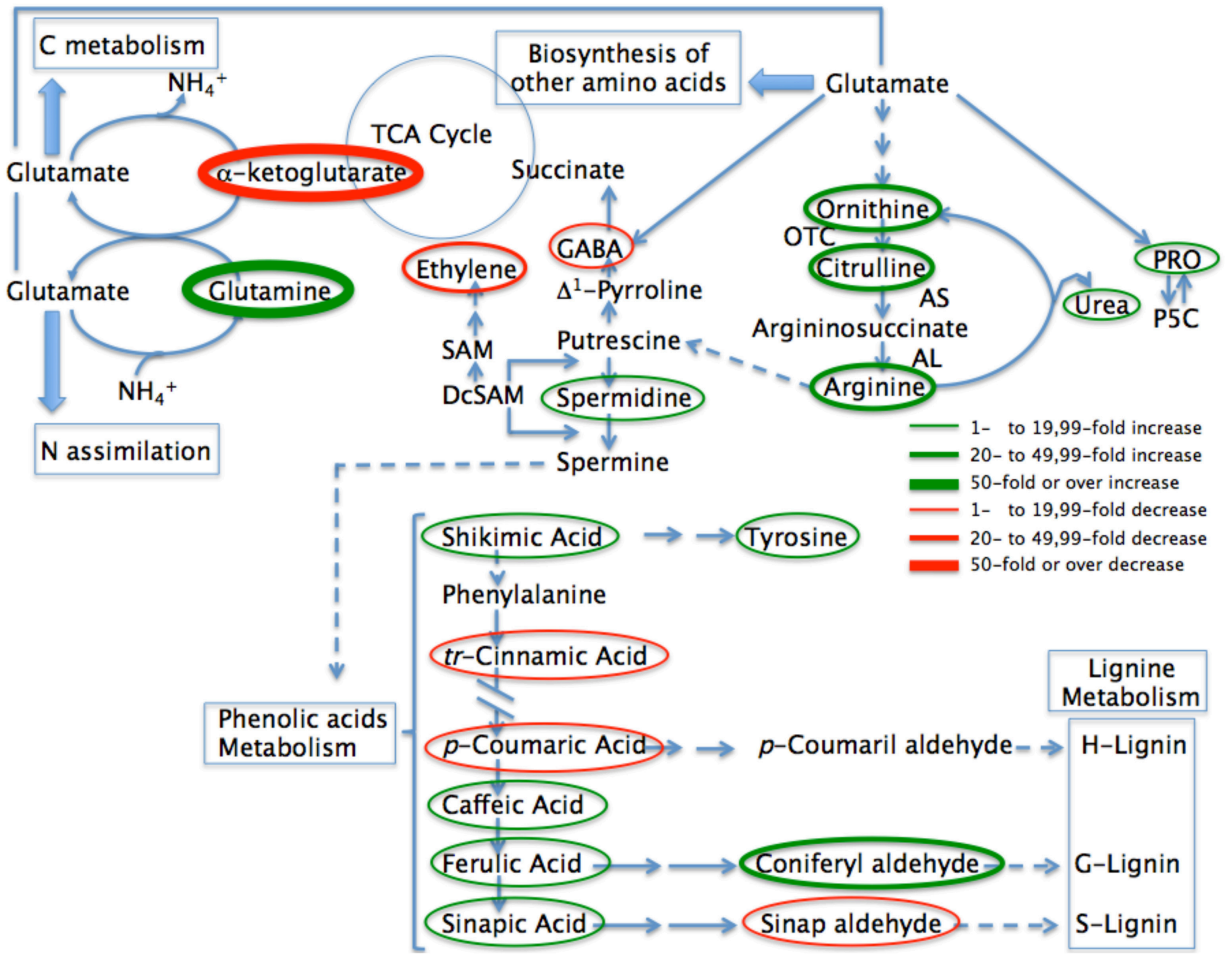
Abbreviated pathway for the biosynthesis of metabolites most affected in pepper plants when inoculated with *Microbacterium* sp. 3J1 during drought compared with uninoculated plants. Metabolites where the concentration is reduced as the result of microbial inoculation are circled in red while those where the concentration increases are circled in green. Thin circles represent changes between 1 and 19.99-fold, medium size circles represent changes from 20- to 49.99-fold in concentration, while thicker circles represent changes over 50-fold in concentration. The most likely pathways are represented. TCA, Tricaboxylic acid; PRO, proline; P5C, proline 5 carboxylate; OTC, ornithine transcarbamoylase; AS, argininosuccinate synthase; AL, argininosuccinate lyase; SAM, *S*-adenosylmethionine; DcSAM, decarboxilated S-adenosylmethionine.

The biosynthesis of proteins is reduced during drought, and protein hydrolysis may occur, resulting in an increase in soluble nitrogen compounds such as free amino acids (Farooq et al., 2009; Krasensky and Jonak, 2012). The accumulation of different amino acids in plants in response to drought has been extensively described (Bowne et al., 2012). Among these amino acids, accumulation of proline has an important role among the repertoire of accumulated osmoprotectants that enable the maintenance of plant cell turgor (Krasensky and Jonak, 2012). In proline biosynthesis, the main pathway originates with glutamic acid and this occurs both in the cytosol and the chloroplast, however, under drought the re-localization of the Δ^1^-pyrroline-5-carboxylate synthetase (P5CS1) into chloroplasts, results in the chloroplast being the main producer of proline (Figure 5). Zanella et al. (2016) have recently described this close relationship between starch degradation to sustain proline biosynthesis during drought stress (Zanella et al., 2016). Many amino acids were produced in greater quantities in roots of pepper plants inoculated with *Microbacterium* sp. 3J1 under drought-stress condition, particularly glutamine being the most affected metabolite in response to the presence of the endophyte under stress (See Table 1). Glutamine increased over 450-fold under dry conditions when *Microbacterium* sp. 3J1 was present, followed by valine, the ornitine-citruline-arginine complex, and asparagine with nearly a 50-, 40-, and 28-fold increase respectively (Figure 5). Other amino acids were found to increase in the plant when the microorganism was present such as histidine, alanine, isoleucine, leucine, proline, arginine, or tyrosine with increases between 6- and 1.6-fold. Amino acids of the aromatic family like histidine, or tyrosine have been described to take part in maize and wheat plants response to drought (Harrigan et al., 2007; Less and Galili, 2008; Bowne et al., 2012; Witt et al., 2012; Galili et al., 2016). While histidine has been described as part of the response to abiotic stress (Harrigan et al., 2007), tyrosine is synthesized through the shikimate pathway. In this respect we also found an increase in the presence of shikimate in the plant inoculated with *Microbacterium* sp. 3J1 (see below in organic acids). Tyrosine acts as substrate for the production of a wide range of secondary metabolites, including reactive oxygen species (ROS) scavengers (Less and Galili, 2008; Gill and Tuteja, 2010). The increase in the production of histidine and tyrosine in bacterized pepper plants under drought conditions is consistent with results of Timothy plants inoculated with *B. subtilis* under similar conditions described by Gagné-Bourque et al. (2016). We did not observe a substantial increase in phenylalanine concentrations in bacterized pepper plants as was observed for Timothy plants by Gagné-Bourque et al. (2016). This may reflect a different physiology of the plants concerned, of the microorganisms or of the interaction plant-microorganism established by both consortia to remove toxic ROS (Chaves et al., 2003; Gagné-Bourque et al., 2016). The increase in branched chain amino acid families such as valine, leucine and isoleucine in pepper roots inoculated with *Microbacterium* sp. 3J1, also agrees with a similar effect on *B. subtilis*-bacterized Timothy plants (Gagné-Bourque et al., 2016). The results we observed for the production of branched amino acids by *Microbacterium* sp. 3J1 inoculated pepper plants support the idea that these amino acids may play an active role in tolerance to drought as previously reported for wheat and peas (Charlton et al., 2008; Bowne et al., 2012). Taylor et al. (2004) stated that branched amino acids have also been proposed to act as osmolytes for *Arabidopsis* and Timothy during drought (Taylor et al., 2004; Joshi and Jander, 2009; Gagné-Bourque et al., 2016). With regard to the aspartate family of amino acid, it is worth mentioning that aspartic acid, asparagine, threonine and lysine have been reported to accumulate in different plant tissues under stress (Barnett and Naylor, 1966; Kusaka et al., 2005; Lea and Azevedo, 2007). We specially note the high increase in asparagine (nearly 28-fold increase) in roots of *Microbacterium* sp. 3J1-bacterized pepper plants under drought which is in agreement with the report by Gagné-Bourque et al. (2016) for *B. subtilis*-inoculated Timothy plants and also with Tall Fescue colonized by the fungal endophyte *Neotyphodium coenophialum* by Nagabhyru et al. (2013) and Gagné-Bourque et al. (2016).

Within the glutamate family of amino acids, proline has been the most reported compatible xero- and osmo-protectant (jointly with glycine-betaine) as well as being a metabolite involved in stress- signaling that is accumulated in a wide range of plants in response to different environmental stresses (Chaves et al., 2003; Julca et al., 2012; Krasensky and Jonak, 2012). The increase in proline levels has been described in pepper plants in response to other abiotic stresses such as salinity by Penella et al. (2016). These authors suggested that the increase in proline levels could protect enzymatic stability from salt-triggered damage, preserving its photosynthetic performance (Penella et al., 2016). We observed previously both the increase in proline and a better photosynthetic performance in pepper plants inoculated with the 3J1 strain, although we cannot determine a cause-effect connection between both observations. The increase in proline in plants is normally associated with the accumulation of its precursor glutamic acid, ornithine and arginine (Ashraf and Foolad, 2007). The presence of *Microbacterium* sp. 3J1 in pepper plants resulted in an even greater accumulation of proline along with some of their precursors such as arginine with around 2-fold increases. However, the highest increase in all metabolites in the plant due to the presence of the microorganism was a different amino acid, glutamine as previously mentioned. The extraordinary increase in glutamine (above 455-fold increase) in the roots due to the presence of *Microbacterium* sp. 3J1 seems to be related to a general interaction of the microorganism with one of the major interactive pathways for carbon (C) and nitrogen (N) assimilation and partitioning. Ammonium ion, derived either from nitrogen assimilation or from photorespiration, is incorporated into glutamine by a reaction catalyzed by glutamine synthase (GS), and glutamine is further converted into glutamate catalyzed by glutamate synthase (GOGAT). The glutamine to glutamate and to proline, ornithine, arginine, and polyamines group pathways involve a large number of interactions in the physiology of the plant. These metabolic routes are the main source for putrescine, spermidine, and spermine biosynthesis, required for the integrity of the cell membranes, cell survival, and growth due to their interactions with the nucleic acids during transcription and translation (Kusano et al., 2007; Minocha et al., 2014). In addition, these pathways are involved in the production of the non-protein aminoacid 4-aminobutyric acid (GABA) and therefore as a phyto-hormone this plays a major role in the plant for several cellular functions (Shelp et al., 2012a,b). In turn the interactions of these polyamides with hydroxycinnamic acids, fatty acids or alkaloids are the basis of the stress-response of the plant (Flores and Filner, 1985; Bagni and Tassoni, 2001; Martin-Tanguy, 2001; Subramanian et al., 2015). Despite the increase in the proline concentration under drought in plants, the presence of the *Microbacterium* sp. 3J1 seems to produce even higher accumulation of this amino acid. Gagné-Bourque et al. (2016) also described a general increase in amino acids in timothy plants when inoculated with the RDTE *B. subtilis* B26 strain. However, we observed that the presence of *Microbacterium* sp. 3J1 also produced a reduction on some amino acids especially for cystein, serine, S-*methy*l-cysteine (with nearly a 24-, 18-, and 10-fold reduction respectively). Other amino acids decreased in concentration in the plant in response to the presence of the microorganism, such as methionine, histidine, aspartate, and more significantly GABA which was reduced between ~6- and 1.5-fold due to the presence of the bacterium. However, under interaction with the endosymbiont, signaling pathways, and amendments in efficiency allow the plant not to produce and accumulate those molecules. These results were surprising since GABA concentration normally increases in drought-subjected tissues and is considered to provide an important role in sensing the stress for an appropriate physiological response from the plant (Kinnersley and Turano, 2000). Therefore, the decrease in GABA in pepper plants subjected to drought due to the presence of the 3J1 strain seems to respond to the restoration of normal conditions where GABA is not needed to the same level as in absence of the microorganism. In addition to a decrease of GABA we also observed a 10-fold decrease in β-aminobutyric acid (BABA), an isomer form of GABA, also involved in drought protection (Jakab et al., 2005).

Regulation of GABA in plants is complex as there are several enzymes associated to the GABA anabolism, and this is especially due to the compartmentation of the GABA metabolism in the cell (Shelp et al., 2012b). While the production of arginine from glutamate (via ornithine) is a reversible pathway as is the production of proline from glutamate or from ornithine, the production of putrescine is a branched and irreversible pathway where ornithine and arginine are used as substrates (Figure 5) (Shelp et al., 2012b; Majumdar et al., 2016). The increase in spermidine and the reduction on GABA in the plant roots due to the presence of *Microbacterium* sp. 3J1 point to a repression of the enzyme glutamate decarboxylase by the microorganism that alters the general interactive pathways for carbon and nitrogen assimilation and partitioning, resulting in the increase on some polyamine biosynthesis via induction of the polyamine oxidase activity.

##### Polyamines

Polyamines are small molecules of aliphatic polycationic nature with variable hydro- carbon chains and two or more amino groups (Takahashi and Kakehi, 2010). Although polyamines were initially thought to have only a structural role, later studies have identified their important role in key processes such as cell division, differentiation, transcriptional regulation, and translation (Tiburcio et al., 2014). The most abundant polyamines in plants are putrescine, spermidine, and spermine and they have been shown to be required for plant stress tolerance by genetic studies (Alcazar et al., 2010). Both biosynthetic and catabolic pathways of polyamines are well-characterized (Martin-Tanguy, 2001; Kusano et al., 2008). Putrescine is synthesized from arginine and/or ornithine by arginine decarboxylase (ADC; EC 4.1.1.19) and ornithine decarboxylase (ODC; EC 4.1.1.17), respectively. Putrescine can be converted to spermidine by spermidine synthase (SPDS, EC 2.5.1.16), and spermidine can be transformed into spermine by spermidine synthase (SPMS, EC 2.5.1.22). However, for the biosynthesis of spermidine and spermine, S-adenosyl-L-methionine is required as an aminopropyl donor. This reaction is catalyzed by S-adenosylmethionine decarboxylase (SAMDC; EC 4.1.1.50) using S-adenosyl-methionine as a substrate which leads to reducing the concentration of substrate for ethylene production (Figure 5). Such antagonism between synthesis of higher polyamines and ethylene is reinforced since the enzymes involved in their synthesis are under feedback control by their end products (Moschou et al., 2008). In this study pepper plants inoculated with *Microbacterium* sp. 3J1 gave a much higher concentration of spermidine with an over 7-fold increase. Therefore, the marked reduction in ethylene production in inoculated plants may respond to a double effect due to the ACC-deaminase activity and due to the reduction on SAM as an ethylene substrate when the microorganism is present. This reduction on the SAM content in the plant also seems to be a consequence of the repression of its biosynthetic pathway, since despite the increase in the citrate cycle intermediates, a decrease in the concentration of aspartate and methionine (intermediates of the SAM biosynthetic pathway) was observed when *Microbacterium* sp. 3J1 was present, with 1.5- and nearly 6-fold decrease respectively.

Subramanian et al. (2015) described the increase in proline and glutamic acid as precursors of ornithine and arginine synthesis in wheat plants due to the presence of hessian fly larvae due to the production of nutrients to feed the parasitic larvae. This increase of ornithine and arginine in turn entered the pathway for polyamine biosynthesis, leading to higher levels of free polyamines putrescine, spermidine, and spermine pointing to a usurpation of the polyamine biosynthesis of the plant by the parasite to acquire nutrients for the growth and development of the larvae (Subramanian et al., 2015). A similar effect may be happening in pepper plants inoculated with *Microbacterium* sp. 3J1 where a higher concentration of nutrients may be more accessible to the microorganism by the overproduction of spermidine. These nutrients may include the carbon sources used for the production of other osmoprotectants such as trehalose. This confirms a report where exogenous application of spermidine improved the drought tolerance of white clover by increasing the levels of soluble carbohydrates such as sucrose, fructose, and sorbitol as well as dehydrin synthesis (Li et al., 2015). Nonetheless the role of spermidine in drought tolerance in plants has also been associated to the upregulation of some gene expression coding for important enzymes involved in the antioxidant response including superoxide dismutase, or catalase among others. Spermidine has also been described as enhancing the glyoxalase system, which reduced methyglyoxal toxicity, a stress-induced compound (Rouphael et al., 2016; Sánchez-Rodríguez et al., 2016). Besides this, spermidine has been shown to enhance the activation of important enzymes involved in the production of nitric oxide in the cell, such as the nitrate reductase and nitric oxide synthase. Both nitric oxide and spermidine-induced antioxidant machinery trigger the signaling cascade to enhance the drought stress tolerance in a nitric oxide-mediated process (Peng et al., 2016). In addition, nitric oxide also modulates abscisic acid signaling during drought, resulting in stomatal closure in a process jointly mediated by abscisic acid and nitric oxide (Wang et al., 2015). Therefore, the better photosynthetic efficiency we have observed in inoculated pepper plants maybe the result of stomata closure by the combined production of abscisic acid directly produced by the microorganism and indirectly produced by the plant in a spermidine-dependent process that is also mediated by the presence of the *Microbacterium* sp. 3J1.

The most described form of polyamines in plants are in free amine forms but plant N-acyltransferases can catalyze their conjugated forms associated with phenolic acids such as hydroxycinnamic acids forming hydroxycinnamic acid amides. Although previously polyamine conjugates were considered to be inactive forms, it is becoming clear that these conjugates are essential for development and for biotic interactions of the plant (Tiburcio et al., 2014).

##### Phenolic acids

Nakabayashi et al. (2014) described the role of phenolic acids as a significant group of non-enzymatic antioxidants involved in the response to drought and in drought-tolerance (Nakabayashi et al., 2014). The role of phenolic acids as high antioxidants depends on their structure including the presence of a large number of hydroxyl groups (Arora et al., 1998). The antioxidant activity of these phenolic compounds includes the reduction of the damaging effects of free radicals, and therefore the protection of enzymatic complexes of the cytoplasm and chloroplasts in mesophyll and epidermal cells (Agati et al., 2007). These compounds can filter UV-A and UV-B radiation absorbing most of the high-energy UV radiation and, in the case of ferulic acid, preventing photo-inhibitory injuries to the photosynthetic apparatus during drought (Hura et al., 2012). In addition, phenolic compounds can act as energy dissipaters and outlets for photo-assimilates if there is energetic imbalance under stress conditions by transmitting or absorbing the energy from other molecules (Hernández and Van Breusegem, 2010). Also, when cell wall becomes saturated with phenolic compounds there is an improvement in the wall's compactness, tightness and hydrophobic character, and therefore a limitation in the movement of water from symplast into apoplast and within the apoplast (Hura et al., 2017). Phenolic compounds also have a key role in cell wall formation, where in the process for biosynthesis of phenylpropanoid/lignin, the cinnamic acid is transformed into *p*-coumaric acid. The presence of *Microbacterium* sp. 3J1 in pepper plants produced an increase in the concentration of ferulic acid and derivatives such as sinapic acid, caffeic, and most notably on coniferyl aldehyde of nearly 2-, 3-, 4-, and 20-folds increase respectively. Increase in ferulic acid and its derivatives have previously been reported during drought in wheat, triticale and barley (Hura et al., 2009, 2012; Piasecka et al., 2017). The increase in ferulic acid concentration has also being reported in plant reactions to different stress conditions including UV radiation (Hura et al., 2012), cold shock in *A. thaliana* (Kaplan et al., 2004), and in response to fungal diseases in barley (Hendawey et al., 2014). In addition, the increase in ferulic acid has been associated with increased drought tolerance in barley (Li et al., 2013). Interestingly we observed higher than 9- and 1.8-fold decrease in *p*-coumaric acid and cinnamic acid respectively in pepper plants when *Microbacterium* sp. 3J1 was present. The fact that the presence of the microorganism results in the reduction of the intermediates for the production of H- and S-types of lignin while also accumulating the intermediates for the production of G-lignin, points to a reordering in the composition of lignin (Figure 5). Research based in the elimination of genes involved in the production on G-lignin in xylem resulted in the accumulation of cinnamic derivatives and the collapse of water-transporting cells under the negative pressure generated by transpiration (Piquemal et al., 1998; Jones et al., 2001; Franke et al., 2002). Therefore, we suggest that the increase in G-lignin substrates and the reduction on H- and S-lignin substrates produced in presence of *Microbacterium* sp. 3J1 may result in a better-suited xylem with fitter water-transporting cells.

These changes in the substrate for lignin production could also affect the synthesis of a plethora of additional chemicals of relevance in plant such strigolactones (Winkel-Shirley, 2001; Umezawa, 2010; Fraser and Chapple, 2011). There are several research reports pointing to the fact that the production of *p*-coumaric acid by the plant influences the biodiversity of the microbial population in the rhizosphere (Zhou and Wu, 2012). Therefore, we cannot disregard that the reduction of the *p*-coumaric acid we observed in pepper plants when inoculated with *Microbacterium* s. 3J1 may have an effect on the rest of the microbial population in the roots.

##### Nucleotides

Pepper plants inoculated with *Microbacterium* sp. 3J1 also increased the production of certain nucleotides such as thymine or uracil (by 2.56- and 4.57-fold), or their substrates such as adenosine (by 2.45-fold) that may help to increase DNA production in the plant. We have recently described the role of the DNA molecule in the protection of biomolecules from damage produced by drought (García-Fontana et al., 2016). Therefore, we do not discard the production of DNA by the plant in presence of the microorganism as a defensive system from the deleterious damage produced by drought.

##### Linking the C and N metabolism through glutamine and α-ketoglutarate

The changes in metabolites of pepper plants during drought due to the presence of *Microbacterium* sp. 3J1 seems to alter the carbon and nitrogen metabolism. In other microorganisms like *B. subtilis* the conversion of glutamate to glutamine and the transformation of glutamate to α-ketoglutarate provides the central link between carbon and nitrogen metabolism (Commichau et al., 2007). Interestingly, two of the metabolites most affected by the presence of *Microbacterium* 3J1 in pepper plants subjected to drought are glutamine (with an over 450-increase being the biggest increase) and α-ketoglutarate (with an over 70-fold decrease, being the biggest decrease in the metabolome profile) (Figure 5). These results suggest that the changes in the metabolites observed in the plant might be determined by the bacterial metabolism. To find out a possible correlation in the metabolism of the microorganism when subjected to drought, a metabolome study was performed in *Microbacterium* sp. 3J1 under normal and drought conditions independently of the presence of the plant (Table 2). A concomitant increase in the carbon metabolism was observed with a general increase of the substrates of the glycolytic pathway, including glucose-6-P, fructose-6-P, glycerate-3-P, and pyruvate with nearly 11-, 18-, 3-, and 2-fold increase respectively. This increase in the glycolytic intermediates when 3J1 strain is colonizing pepper plant subjected to drought might also contribute to the important increase in trehalose concentration with nearly 85-fold increase. Intriguingly, a near 2.5-fold reduction on phosphoenolpyruvate (PEP) was observed in the inoculated plant. Phosphoenolpyruvate is an intermediate of the glycolytic pathway which might be linked to the near 1.6-fold reduction on erythrose-4-P observed, both of them involved in the production of shikimate via 3-deoxy-D-arabinoheptulosonate-7-P, for the production of salicylic acid or auxin.

**Table 2A:** Metabolites reduced in *Microbacterium* sp. 3J1 in response to drought.

**Metabolite**	**Ratio**	**Type**
Alanine	16.34	
Leucine	16.46	
Glycine	58.73	
Norleucine	5.19	
Serine	2.58	
Threonine	43.33	
S-Methilcysteine	2.00	
Thymine	73.29	
Homoserine	3.83	
Glutamine	5.77	
Methionine	6.75	
Cysteine	1.95	
Cytosine	7.00	
Hydroxyproline	6.94	
Phenylalanine	3.90	
Asparagine	7.37	
Spermidine	2.00	
L-Cystathionine	2.59	
1-Metil-L-Histidina	2.17	
Ethanolamine	5.87	
Maleinic acid	6.98	
Cinnamic acid	7.78	
p-Coumaric acid	17.26	
Sinapic acid	12.48	
Gluconate	1.74	
α-Ketocaproate	1.83	
Succinate	7.09	
Malate	5.60	
4-Aminobutirate (GABA)	22.94	
α-Ketoglutarate	5.85	
Glutamate	4.26	
cis-aconitate	3.01	
Citrate	2.29	
2-Hydroxyglutarate	3.23	
2-Methylcitrate	13.92	
Urea	3.65	
Uracil	2.22	
myo-Inositol	13.00	
Orcinol	6.056	
Pinitol	2.59	
Siringaldehyde	16.04	
Ribulose-5-P	1.77	
Frutose-6-P	8.00	
Xylose	2.37	
Arabinose	12.84	
Ribose	8.86	

**Table 2B:** Metabolites concentrated in *Microbacterium* sp. 3J1 in response to drought.

**Metabolite**	**Ratio**	**Type**
Valine	3.43	
Isoleucine	9.71	
Proline	5.19	
Homocysteine	4.52	
Ornithine-Citruline-Arginine	8.60	
Tyrosine	1.52	
Histidine	12.72	
Lysine	19.14	
Tryptophan	10.65	
Cystine	1.75	
N-Acetylglucosamine	4.51	
ß-Aminoisobutyric acid	1.57	
Pantothenic acid	13.54	
Glucoronic acid	6.32	
3-Hydroxy-4-Methoxy-Cinnamic acid	4.81	
Dihydrocaffeic acid	14.53	
Pyruvate	7.10	
Glycerate	9.71	
Fumarate	9.71	
Phosphoenolpyruvate (PEP)	9.71	
Dihydroacteonphosphate (DHAP)	9.71	
Isocitrate	6.17	
2-Methylcitrate	6.63	
Gluconate-1,5-lactone	20.03	
Adenine	15.31	
Adenosine	5.59	
Mannitol	8.50	
trans-Chalcone	17.72	
Conilferyaldehyde	7.45	
Glycerate-2-P	3.51	
Glycerate-3-P	5.13	
Erythrose-4-P	9.71	
Glucose-6-P	4.43	
Glucose-6-P	8.96	
Ribose-5-P	4.59	
myo-Inositol-P	4.30	
Rhamnose	9.71	
Fructose	18.77	
Glucose	8.09	
Galactose	3.52	
Sucrose	15.86	
Maltose	9.71	
Melibiose	7.47	
Trehalose	6.27	

##### Metabolome of microbacterium sp. 3J1 in response to drought

To study the metabolomic response of *Microbacterium* sp. 3J1 to drought two cultures of the microorganism were grown at 5 and 50% PEG 6.000 to simulate a normally hydrated soil and a drought-subjected soils respectively. The metabolome was analyzed on the cellular content to distinguish between intracellular-produced molecules that significantly increased or decreased in concentration in response to drought. A general coincidence on the metabolites that increased and decreased in the pepper plant when inoculated with the microbial strain was observed for *Microbacterium* sp. 3J1 when subjected to drought with 27 metabolites that significantly increased in the microorganism in response to drought that also increased in plants subjected to drought when inoculated with this microorganism (Table 2). This general coincidence points to a shared mechanism to protect both types of cells (plant and microbial cells) from drought or to a net contribution of the microorganism in the plant physiology by overproduction of the identified metabolites. We also identified nine metabolites that increased in *Microbacterium* sp. 3J1 in response to drought that were not identified in inoculated drought affected plants. These nine metabolites included gluconate-1,5-lactone, lysine, sucrose, adenine, pantothenic acid, fumarate, dihydroacetonephosphate (DHAP), rhamnose, homocysteine, and glycerate-2P, (with increases ranging from 20.03-fold to 3.51-fold) that either were not synthetized or exported by the microorganism when inside the plant or that the net contribution of the microorganism was not sufficient to be detected in the plant. The most surprising results responded to five metabolites that increased in the bacterium in response to drought but decreased in plants subjected to drought when inoculated with the same bacteria. These compounds were histidine (12.72-fold), β-aminobutyric acid (1.5-fold increase), glycerate (9.7-fold increase), PEP (9.7-fold increase), and erythrose-4P (9.7-fold increase). The differential production by the microorganisms compared to the microbial-inoculated plant of these metabolites may be explained by the use of them by bacteria as a mechanism of self-regulation (necessary compounds for stress management in the strain). Or alternatively that under the interaction conditions the regulation of processes makes it unnecessary to synthesize (as molecular signals and intermediary for histidine and β-aminobutyric acid) and accumulate these compounds (as osmoregulator or energy supplies for PEP and erythrose-4P). We have demonstrated that some metabolites that increased in both, microorganism subjected to drought and drying plants inoculated with the microorganism, such as trehalose, are the result of the re-configuring of the plant metabolism by the microorganism (Garcia-Fontana et al., unpublished results). Therefore, we speculate that a general re-configuring of the pepper plant metabolism might be occurring when inoculated by *Microbacterium* sp. 3J1 during drought conditions that promotes better survival of the plant.

We also found a decrease in some metabolites produced by *Microbacterium* sp. 3J1 in response to drought (see Table 2). Among them the most remarkable reduction corresponded to thymine, glycine, threonine, GABA, and *p*-coumaric acid (with over 73-, 58-, 43-, 22-, and 17-fold decrease respectively).

It is surprising that among the metabolites produced by the microorganism in absence of the plant we find phytohormones, and lignin intermediates that might point to a tight ecological relationship of the microorganism with the plant. The fact that the plant overproduced *p*-coumaric acid in drying conditions when inoculated with the endosymbiont while the microorganism reduced the concentration of the same metabolite during drying conditions might point to a way by which the microorganism can compete with other microorganisms in the rhizosphere under non-stressing conditions. However the stringent conditions of water scarcity may provide a competitive advantage to the desiccation-tolerant *Microbacterium* sp. 3J1 and therefore there is no need to produce *p*-coumaric acid in order to compete with other microorganisms.

Once inside the plant the microorganism seems to re-route its metabolism especially regarding the carbon and nitrogen balance, since concentrations of glutamine and α-ketoglutarate were slightly reduced (by ~5.8-fold in both cases) in response to drought in the free-form of the microorganism, while glutamine strongly increased in plants subjected to drought when the microorganism was present.

## Conclusion

To our knowledge, this is the first study on comparative metabolomics between a RDTE and the pepper plant it colonizes under water stress conditions. Here we demonstrate the endophyte nature of *Microbacterium* sp. 3J1 and the protection against drought resulting from this symbiotic relationship with the plant. We observed better agronomical traits such as higher relative water content and higher photosynthetic efficiency in inoculated plants after 7, 14, 20, and 33 days of drought stress. Our results indicate that production of osmo-protectants and antioxidants may collectively mediate enhanced resistance to water deficit in pepper plants when inoculated with *Microbacterium* sp. 3J1. These results clearly show that strain 3J1 improved pepper plant responses to drought stress by increasing the accumulation of mostly acquired and some inducible metabolites associated with drought protection compared to non-inoculated plants. The presence of the endophyte resulted in increases in sugars such as trehalose, melibiose, fructose, and glucose under drought conditions which were directly linked to the presence of *Microbacterium* sp. 3J1, and were also produced by the microorganism to protect itself from drought. The colonization of the plant by the microorganism resulted in the accumulation of amino acids that were produced at greater quantities under water stress with notable increases in glutamine, ornithine, citrulline, and arginine. With an increase in glutamine in the range of the decrease detected for α-ketoglutarate pointing to a general effect on the nitrogen and carbon metabolism balance mediated by the presence of *Microbacterium* sp. 3J1. The increase in spermidine accompanied by the decrease in GABA and ethylene points to a re-routing of the polyamines biosynthesis for the modification of the lignin substrates resulting in different chemical organization. Such alteration in lignin content seems to allow the release of the sugars, and to facilitate the transit of water and nutrients as well as the xero- and osmo-protectants produced by the microorganism to counterbalance the oxidative damage produced by the closure of the stomata.

## Author contributions

JV, KN, DD, JG-L, and MM designed the experiments and analyzed the data. JV, KN, and DD performed experiments, MM wrote the main manuscript text and JV prepared figures. All authors reviewed the manuscript.

### Conflict of interest statement

The authors declare that the research was conducted in the absence of any commercial or financial relationships that could be construed as a potential conflict of interest.
